# Detection and Strain Typing of Ancient *Mycobacterium leprae* from a Medieval Leprosy Hospital

**DOI:** 10.1371/journal.pone.0062406

**Published:** 2013-04-30

**Authors:** G. Michael Taylor, Katie Tucker, Rachel Butler, Alistair W. G. Pike, Jamie Lewis, Simon Roffey, Philip Marter, Oona Y-C Lee, Houdini H. T. Wu, David E. Minnikin, Gurdyal S. Besra, Pushpendra Singh, Stewart T. Cole, Graham R. Stewart

**Affiliations:** 1 Department of Microbial and Cellular Science, Faculty of Health and Medical Sciences, University of Surrey, Guildford, Surrey, United Kingdom; 2 Department of Archaeology, University of Winchester, Winchester, United Kingdom; 3 Department of Archaeology, University of Southampton, Highfield, Southampton, United Kingdom; 4 Bristol Isotope Group, School of Earth Sciences, University of Bristol, Bristol, United Kingdom; 5 School of Biosciences, University of Birmingham, Edgbaston, Birmingham, United Kingdom; 6 Global Health Institute, École Polytechnique Fédérale de Lausanne, Lausanne, Switzerland; University College Dublin, Ireland

## Abstract

Nine burials excavated from the Magdalen Hill Archaeological Research Project (MHARP) in Winchester, UK, showing skeletal signs of lepromatous leprosy (LL) have been studied using a multidisciplinary approach including osteological, geochemical and biomolecular techniques. DNA from *Mycobacterium leprae* was amplified from all nine skeletons but not from control skeletons devoid of indicative pathology. In several specimens we corroborated the identification of *M. leprae* with detection of mycolic acids specific to the cell wall of *M. leprae* and persistent in the skeletal samples. In five cases, the preservation of the material allowed detailed genotyping using single-nucleotide polymorphism (SNP) and multiple locus variable number tandem repeat analysis (MLVA). Three of the five cases proved to be infected with SNP type 3I-1, ancestral to contemporary *M. leprae* isolates found in southern states of America and likely carried by European migrants. From the remaining two burials we identified, for the first time in the British Isles, the occurrence of SNP type 2F. Stable isotope analysis conducted on tooth enamel taken from two of the type 3I-1 and one of the type 2F remains revealed that all three individuals had probably spent their formative years in the Winchester area. Previously, type 2F has been implicated as the precursor strain that migrated from the Middle East to India and South-East Asia, subsequently evolving to type 1 strains. Thus we show that type 2F had also spread westwards to Britain by the early medieval period.

## Introduction

Leprosy is a chronic granulomatous disease caused by *Mycobacterium leprae*, first identified as the causative pathogen by the Norwegian physician Armauer Hansen, in 1873. In leprosy there is a spectrum of clinical presentations dependent upon the cell-mediated immune (CMI) response of the host. Tuberculoid leprosy (TL) is associated with an effective CMI response and few mycobacteria whereas lepromatous leprosy (LL) is characterised by poor CMI and widespread lesions with numerous mycobacteria. A number of intermediate conditions are recognized; borderline tuberculoid leprosy (BT) and borderline lepromatous (BL) leprosy. A recent WHO classification scheme recognises two simplified categories of either paucibacillary or multibacillary forms of leprosy [1]. Any form of leprosy may affect the skeleton; even paucibacillary forms resulting in loss of pain sensation with digital contracture, volar grooving or osteomyelitis complicating an overlying skin infection [2]. The more typical lesions of the hands, feet and rhino-maxillary area [3] result from multibacillary states at the severe end of the spectrum.

*Mycobacterium leprae* is a clonal organism, with limited genetic variation between strains. Although sequencing and comparative genomics of four main strains has revealed a total of 215 polymorphic sites, primarily of single nucleotide polymorphisms (SNPs) this is against a background of high overall genome conservation (>99.99% homology). Study of the phylogenetically informative SNPs in *M. leprae* isolates from different geographical regions has allowed the original spread of leprosy to be retraced back to a likely origin in East Africa [4]. There remains some doubt as to how long leprosy has been a human pathogen. The leprosy bacillus may have moved out of Africa and around the world with early human migrations around 100 K years BP, or may have encountered different populations much later in prehistory, with documented spread in the later historical period due to military adventures, colonialism and trade. As genetic data for modern and particularly archaeological strains of leprosy are accumulated, an increasingly detailed understanding of the rise and fall of leprosy across the globe is being revealed.

Leprosy was present in the British Isles by the 4^th^ century AD with palaeopathological evidence described from the Romano-British site of Poundbury Camp in Dorset [5] and later 6^th^ century AD Anglo-Saxon burials from Beckford, Hereford and Worcester [6] and also Tean in the Scilly Isles [7]. However, it is later on in the high middle ages, between the 11^th^ and 14th centuries, when the disease was probably at its height in southern Britain. A number of *leprosaria* were established during this period to serve both the spiritual and physical needs of sufferers. The establishment of *leprosaria* in Britain is usually thought of as a post-Norman conquest phenomenon in response to the rise in the disease along with increases in population size and the development of towns. The number of known foundations of *leprosaria* numbered more than 300 before 1350 AD, whereas after this period and up to the dissolution of the monasteries in 1538, the number of new foundations declined to around two dozen [8]. Thereafter, surviving *leprosaria* were often adapted for other uses, such as almshouses.

There has been much debate about the accuracy of medical diagnosis of the inmates of *leprosaria* and also for the reasons for the decline in leprosy, which may have been multifactorial. There is little information available on *leprosaria* and few have been excavated. A notable exception is the house at Chichester, of St. James and St. Mary Magdalene founded in 1118 and located to the east of the medieval town. Here, around 22% of the 400 recovered adult burials showed evidence of LL [9]. Excavations at St. John’s Timberhill in Norwich suggest a frequency of leprosy in around 20% of the burials [10], [11]. Confirmation of *M. leprae* pathogen DNA and limited strain genotyping has been applied to several cases from this site [12].

The material examined in the present study comes from the site of St. Mary Magdalen, situated about one mile to the east of Winchester in Hampshire. This is thought to be an early Norman foundation, established sometime between 1070 and 1090 AD. It was re-founded sometime after 1148 by Henry de Blois, Bishop of Winchester from 1129 until his death in 1171. The hospital for those afflicted with leprosy survived the Reformation but by the end of the 16^th^ century the masonry-built built Infirmary was demolished to make way for brick-built almshouses. These in turn were demolished in the 1780’s [13], [14]. The site was the subject of a televised Time Team dig in 2000. Survey and current re-evaluation of the site began in 2007 and has revealed evidence for several structures including a Chapel, Master’s lodge, Infirmary, gatehouse and ancillary buildings. To date, 56 burials have been excavated, 38 of these from the early northern cemetery [15]. An unusually high proportion (86% of those in the cemeteries) show skeletal evidence of LL. Thus these samples offer an exceptional opportunity to perform a palaeomicrobiological assessment of *M. leprae* from a time period when very little is known about the phylogenetic basis of leprosy in Northern Europe. The current study uses a multi-disciplinary approach to study the human remains and the strains of *M. leprae* responsible for disease at this place and time in the medieval period.

## Materials and Methods

### Osteology

**Figure 1:**
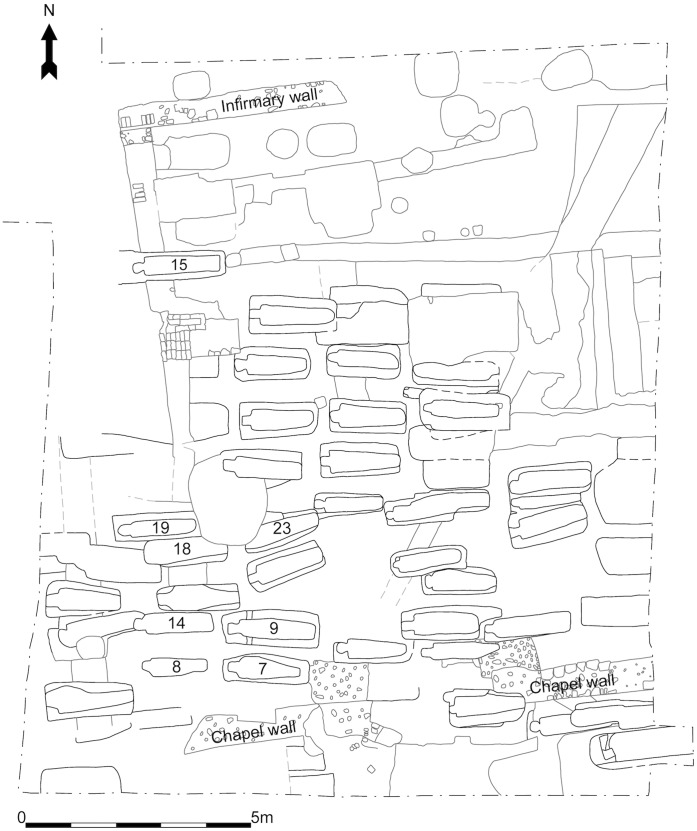
Schematic plan of the St. Mary Magdalen hill dig site, looking north. The north wall of the medieval chapel is in the foreground whilst the remains of the medieval infirmary can be seen to the north. The northern cemetery can be seen depicted in the centre of the plan and underlies most of the later medieval phases. Numbers on grave cuts identify individual skeletons examined in the present study.

All necessary permits were obtained for the described field studies, including a licence (-0070) to exhume and retain human remains, provided by the Ministry of Justice, 102 Petty France London SW1H 9AJ. A schematic plan of the dig site, including the northern cemetery, is shown in Figure 1. All of the skeletons that are the subject of this paper were excavated by hand from sealed contexts and were all (with the exception of Sk12, which was commingled with Sk13) from single chalk-cut graves with only very limited evidence for truncation or disturbance. Data on the grave type and body position of each individual can be found in Table 1. As the possibility of finding individuals with skeletal evidence for leprosy was anticipated prior to excavation, they were also subject to one hundred percent sampling of the grave fills, which were then hand-sorted. This allowed for near complete retrieval of the small bones of the hands and feet, which are invaluable for the correct diagnosis of leprosy, particularly in its early stages.

**Table 1:** Body position and grave type of the analysed skeletons (R = right, L = left).

Skeleton number	Context number	Grave type	Body position
1	15/041	Rectangular grave, cut through earlier graves; evidencefor a nailed wooden coffin.	Supine, extended, hands on the pelvis.
2	15/050	Sub-rectangular grave, cut through cemetery soil	Supine, extended, upper limbs by the sides of the torso.
7	14/227	Rectangular chalk-cut grave with anthropomorphicinner cut.	Supine, extended, L upper limb crossed over the abdomen, R upper limb flexed at the elbow with the hand on the R shoulder.
8	14/179	Rectangular chalk-cut grave with an anthropomorphicinner cut.	Supine, extended, evidence for bone-tumble of torso and displacement of upper limbs and L tibia.
9	14/236	Rectangular chalk-cut grave with an anthropomorphicinner cut.	Supine, extended, upper limbs flexed at the elbow with the hands on the abdomen.
12	15/055	Rectangular chalk-cut and stone-constructed grave witha plaster lining and covered by a Purbeck marble slab.	Disarticulated and commingled with the remains of SK13.
14	14/404	Anthropomorphic chalk-cut grave.	Supine, extended, upper limbs flexed at the elbow and the wrists crossed on the pelvis.
15	14/359	Rectangular chalk-cut grave with an inneranthropomorphic cut; possible heavily decayed woodenobject between the femorae, copper alloy pin recoveredfrom the grave fill sample.	Supine, extended, upper limbs folded across the abdomen with the L hand on the R elbow and the R hand on the L elbow.
18	14/405	Rectangular chalk-cut grave with an inner ledge.	Supine, extended, hands together on the pelvis.
19	14/407	Rectangular chalk-cut grave with an inneranthropomorphic cut; pottery sherds found aboveL side of chest and feet, copper alloy shroud pin recoveredfrom the grave fill sample.	Supine, R lower limb slightly flexed at knee, ankles together, upper limbs flexed at elbow with L hand on R side of pelvis and R hand on L elbow.
23	14/432	Rectangular chalk-cut grave with an inner ledge.	Supine, extended, hands together on L side of abdomen.

The skeletons were osteologically analysed in the laboratory of the Department of Archaeology, University of Winchester, Winchester, UK. Determination of sex in the adult individuals was undertaken using the methods of Phenice [16] for features of the pubic bone, Buikstra and Ubelaker [17] for the greater sciatic notch, and Acsádi and Nemeskéri [18] for features of the cranium and mandible. Individuals were then assigned one of five sex classifications: definite male (M); possible male (? M); indeterminate (?); possible female (? F); definite female (F). Estimation of age in the adult individuals was undertaken using the methods of Brooks and Suchey [19] for the pubic symphysis, and Lovejoy *et al*. [20] for the auricular surface. Estimation of age in the non-adult individuals was undertaken using long bone lengths, dental development and epiphyseal fusion, as outlined in Scheuer and Black 2000 [21]. Individuals were then placed into one of the following age categories, as recently advocated by Falys and Lewis [22] Foetal (up to 40 weeks gestation), neonate (from 40 weeks gestation to 1 month), infant (1 month to 1 year), young child (1–6 years), older child (7–12 years), adolescent (13–18 years), young adult (19–25 years), young middle adult (26–35 years), old middle adult (36–45 years) and mature adult (46+ years).

All evidence for pathology and trauma was documented in detail through the use of descriptions and photographs. Skeletal evidence for leprosy was diagnosed with the aid of Ortner [3], [23] and Lee and Manchester [24], studies that are largely focused on the evidence for the disease amongst the individuals from the medieval site of St. James and St. Mary Magdalene, Chichester. Skeletal changes that could be ascribed to leprosy included the rhino-maxillary syndrome, typified by rounding and thickening of the margins of the nasal aperture, resorption of the nasal spine, porosity and resorption of bone of the palate and nasal floor and of the anterior alveolar margin of the maxilla and, rarely, the mandible. Other characteristic lesions examined for included remodelling of the metacarpals, metatarsals and manual and pedal phalanges, that results in circumferential wasting of the shafts, and “pencil and cup” deformities of metatarsal/metacarpal-phalangeal and interphalangeal joints; flexion contractures of the phalanges of the hand with pressure erosion of the volar metaphyseal area, which result from the neuromuscular degeneration associated with multibacillary leprosy. Moreover, evidence was sought for septic arthritis, reactive diffuse periosteal new bone and well-defined reactive bone formations of the lower limbs and feet, which result from direct infection spreading from the foot or chronic secondary infections, and which occur through damage to the nerves leading to repeated soft-tissue injury and ulceration.

### Biomolecular Study

#### Sampling

Nine skeletons showing osteological signs of leprosy (Table 2) were sampled at the Department of Archaeology, University of Winchester, Winchester, UK. At the time of analysis, 23 skeletons were available for destructive analysis, only two females among them and these did not have strong evidence for leprosy. Bone samples were taken from individuals with the best skeletal evidence for leprosy and best bone preservation. Our sample reflects the fact that the cemetery contained a preponderance of male individuals. Specimens were predominantly taken from areas adjacent to pathological lesions but on occasion also from uninvolved skeletal elements such as rib or cranium. The weights of bone fragments, roughly cuboidal (6–8 mm) in shape, varied from 20–120 mg. Measures to prevent cross-contamination between cases were applied from the outset. To this end, an area of bench was set aside and cleaned before and between cases. Disposable gloves were worn and changed frequently. Disposable scalpels and sterile tubes were used to collect and store bone fragments respectively. To act as controls, two skeletons without signs of leprosy were also sampled (Sk1 and Sk12). In these cases, fragments of bone were taken from around the vomer, as this area typically displays a high mycobacterial burden in LL. Samples from four of these cases (Sk14, Sk18, Sk19 and Sk23) were divided into two equal portions for independent replication at a second centre.

**Table 2:** Summary of osteological data from skeletons examined in the current study.

Sk. No.	Age	Sex	Evidence for leprosy.			
			Hands	Feet	Lower limbs	Cranium
Sk1*	Old middle adult	M	No	No	No	No
Sk2	Old middle adult	M	No	Yes	No	Yes
Sk7	Young middle adult	M	Yes	Yes	Yes	Yes
Sk8	Older child	?(M)	No	No	Yes	Yes
Sk9	Young middle adult	M	Yes	Yes	Yes	No
Sk12*	Old middle adult	M	No	No	No	No
Sk14	Adolescent	?M	No	Yes	Yes	Yes
Sk15	Young adult	M	Yes	Yes	Yes	Yes
Sk18	Adolescent	M	Yes	Yes	Yes	Yes
Sk19	Young middle adult	M	Yes	Yes	Yes	Yes
Sk23	Young middle adult	?F (M)	Yes	Yes	Yes	Absent

* = Skeletons selected as controls.

#### DNA extraction at centre one (AX Building, Division of Microbiology, University of Surrey, Guildford, Surrey, UK)

Bone fragments were ground to a fine powder using sterilised pestles and mortars. The powders were weighed and divided into two approximately equal amounts. One set was extracted immediately for screening using leprosy PCRs, the other was set aside for subsequent genotyping methods. DNA was extracted as previously described [25] using the NucliSens™ extraction kit from bioMériux Limited, Boxtel, The Netherlands. After elution of DNA from the supplied silica, extracts (60 µl) were sub-divided into 2 x 30 µl aliquots and stored in low retention plastic tubes to minimize loss of DNA through repeated freeze-thawing events.

#### DNA extraction at centre two, Global Health Institute, École Polytechnique Fédérale de Lausanne, CH-1015 Lausanne, Switzerland

After sampling, the second centre received approximately 50–60 mg bone powder in sterile eppendorf tubes. The powder was placed in 0.9 ml lysis buffer (10 M GuSCN, in 0.1 M Tris-HCl pH 6.4, containing 0.2 M EDTA and 2.6 gm Triton X-100). Before use, the lysis buffer was filtered through a silica-containing GenElute™ HP Maxiprep binding column (Sigma), to remove any contaminating DNA. The resultant supernatant was again passed through a YM-100 Amicon® filter to retain any molecules larger than 10-kD (such as PCR inhibitor proteins etc). The samples were vortexed and placed on a roller for 20 min. Silica suspension from an agarose gel extraction kit (Roche product number 11 696 505 001) was used to bind any aDNA templates present in the bone samples. These reagents were then processed following the method previously described [25].

#### Polymerase Chain Reaction (PCR) and SNP genotyping at centre one

Both conventional PCR (cPCR) and real-time (RT) PCR platforms were used to screen for and genotype strains of leprosy present in skeletal extracts. The multi-copy element RLEP was used to screen for evidence of *M. leprae* DNA. The method and primer sequences have been previously reported [25]. In the present study, an RT method using the intercalating dye EVAGreen® (Biotium) was used to report product formation. A second RT PCR method for the single copy 18-kD antigen gene was used to confirm presence of leprosy DNA and to assess suitability for further genotyping. A dual-labelled probe, [Joe]ctgcggtcaaaagcccgtcttagccatg [BHQ1], was used to monitor product formation. The sequences of the primers for 18-kD and other PCR methods are shown in Table 3.

**Table 3:** Previously unpublished primers used for screening and genotyping at centre one.

PCR locus	Primers	Method	Amplicon (bp)	Anneal temp C
18-kD antigen	5′-ctaatcgactgttgtttgcgcaac-3′ 5′-gccagcaaccgaaatgttcgga-3′	Probe	114	53
SNP 1,104,235	5′-gtgtggagcacttcaattcgctt-3′ 5′-tgtagtctttagtgtacatcaatccctc-3′	EVAGreen	117	53
SNP 2,751,790	5′-caagccacgcccgtcgggtac-3′ 5′-tgctccgggcgtgaagctggtc-3′	“	115	56
SNP 3,102,787	5′-gtgtggaaaggtggaacgacgat-3′ 5′-cactgattgccttcccgagtc-3′	“	139	52
InDel _17915	5′-accctcgaggacgcgtaacgt-3′ 5′-tagcgttcagtacgatccggaca-3′	“	120	54
SNP 1,527,056	5′-gcgtgaccagcaattcaagcac-3′ 5′-acaccgaatagctgaactcgttgc-3′	“	101	54
Amel X chrom	5′-tgaccagcttggttctawccc-3′ 5′-caratgagraaaccagggttcca-3′	“	290	58
Amel Y chrom	As for Amel X	“	105	58

A series of PCR methods was used to genotype positive extracts. A number of these, used for characterising SNP type 3 strains, have been previously published [26], [27]. Several new methods were developed for sub-typing 3I isolates and for genotyping SNP type 2 strains identified in the present study and these primers are also shown in Table 3.

#### Multiple loci Variable Number Tandem Repeat (VNTR) analysis (MLVA)

MLVA typing was undertaken on all five of the cases selected for genotyping, these being Sk2, Sk7, Sk8, Sk14 and Sk19. Two microsatellite and one minisatellite repeat loci were analysed. These were: (i). ML2344-ML2345 (AGA)20. (ii). ML2172-ML2173 (GTA)9 and (iii). the ML0058c locus also known as 21-3 with a variable number of a 21 bp tandem repeat with the sequence 5′-tgatcaacttgattcctggct-3′. Primer sequences and PCR details have been previously reported [25].

#### Screening of extracts for *Mycobacterium tuberculosis* complex DNA

Extracts were also tested for the presence of *Mycobacterium tuberculosis* (MTB) complex organisms using a real-time PCR method for the IS*1081* repetitive element [28].

#### Sex determination

To obtain some indication of human DNA survival, a sex-determining PCR based on DNA coding for the tooth enamel protein amelogenin was used on selected extracts. Primers were designed to span a deletion in intron 1 of the Y-chromosome homologue, resulting in different sized products from the X and Y chromosomes [29]. The primer sequences are shown in Table 3.

#### PCR at centre one

Both cPCR and RT PCR were performed in a final volume of 25µl, using the Hot start Taq master kit from Qiagen (product 203445). The reactions contained 25 pmol of forward and reverse primers, each in 1µl, 12.5 µl of the 2×kit master mix, 2.5 µl non-acetylated bovine serum albumin (BSA, Sigma B4287), and 1µl of template. The kit provides a magnesium ion concentration of 1.5 mM per reaction. This was supplemented to 2 mM for cPCR and for RT PCR methods using EVAGreen® and to 3 mM MgCl_2_ for RT PCR with the 18-kD and IS*1081* hydrolysis probes. The probes were used at a final concentration of 100 nM, through the addition of 1µl of 2.5µM working stock of probe per 25µl reaction. The volumes were made up to 25 µl with molecular biology grade water (Sigma). After an initial activation step of 14 min at 95°C, 41 cycles of amplification were performed on an Mx3005P RT PCR platform (Agilent Technologies). The thermal profiles consisted of denaturation at 95°C for 10 s, annealing (range 52–60°C) for 30 s and extension at 72°C for 30 s. Fluorescence data was acquired during the extension step in RT PCR runs. Melt analyses was performed automatically at the end of runs monitored with EVAGreen® and dissociation curves studied to identify likely positives.

#### PCR at centre two

Laboratory 2 analyzed four samples (Sk14, Sk18, Sk19 and Sk23) using cPCR in a reaction volume of 20 µl with 45 cycles of amplification on a PeqStar thermal platform. The reaction mixture consisted of Pfu polymerase (Promega), associated buffer (with final concentrations of 20 mM Tris-HCl, pH8.8, 2 mM MgSO_4_, 10 mM KCl, 10 mM NH_4_2SO_4_, 1.0% Triton® X-100 and 1 mg/ml nuclease-free BSA. Primer sequences for the RLEP amplicon (111 bp) were the same as those used at centre 1. The genotyping of these samples was performed following the methods and primers described previously [27], [30].

#### Gel electrophoresis and automated DNA sequencing at centre one

PCR products were run out on 3% agarose gels in a TAE buffer system alongside appropriate DNA size markers (100 bp or 50 bp DNA ladders, Promega) to confirm product identity. Positive samples for SNP or MLVA typing were bulk purified on 3% (wt/vol) low-melting-point agarose (Invitrogen); bands were excised and purified using a Geneclean DNA isolation kit (Cat.No.1001-200 from mpbio.com). Templates were sequenced using both forward and reverse primers by Beckman Coulter Genomics Ltd., Takeley, Essex, UK.

#### Gel electrophoresis and automated DNA sequencing at centre two

PCR products were electrophoresed on 2.5% TAE-agarose gels to check the success of PCR and any non-specific bands. Specific PCR bands were treated at 37°C for 15 min with EXO-SAP-IT® (Exonuclease and Alkaline Shrimp Phosphatase, product 78200/01/02/05/50, USB, Corp., Cleveland, Ohio) to remove any unincorporated dNTPs/primers as per the manufacturer’s instructions, followed by inactivation of the enzyme at 80°C for 15 min. The resulting products were used as templates for BigDye Terminator cycle sequencing and then the sequences were compared as described previously [27], [31].

#### Measures to prevent contamination at centre one

Separate laboratories were used for each of the three main stages of the aDNA analyses, these being **1.** Extraction **2.** PCR amplification and **3.** Post PCR analysis, such as gel electrophoresis and purification of products for sequencing. The pre- and post- PCR laboratories were physically separated and independently equipped with pipettes, fridge-freezers, mixers and bench top centrifuges, disposable plastic ware, filter tips and other reagents dedicated to the project.

Surfaces and equipment in contact with sample tubes (centrifuges, rotors, mixers, etc.) were cleaned before each assay. Two control tubes, comprising reagents less bone powder, were taken through each extraction experiment to ensure reagents were contamination free. Several template blanks were run alongside bone extracts in the PCR machine to screen for random contamination. Positive controls were not included in any of the PCR experiments run at centre one.

#### Measures to prevent contamination at centre two

Physically separate areas and dedicated equipment were used for performing the DNA extraction from bones. After each step, all the equipment was cleaned twice, and gloves changed frequently to avoid contamination or cross contamination. Two extraction blanks and one PCR blank were routinely included. These controls behaved as expected.

The PCR master mix was set up in a vertical laminar flow cabinet in a physically separate area, while addition of the DNA templates was performed in another cabinet. Positive control DNA from modern *M. leprae* was not handled in the same experiments. Whenever positive control samples were used, they were handled in a separate laboratory and only after the analysis of the aDNA samples.

### Lipid Extraction and Mycolic Acid Analysis

Bone sample Sk8 (80 mg) and DNA pre-extracted bone samples Sk2 (50 mg), Sk7 (80 mg), Sk14 (102 mg) and Sk19 (32 mg) were hydrolysed by heating with 30% potassium hydroxide in methanol (2 ml) and toluene (1 ml) at 100°C overnight [26], [32]–[34]. In parallel, standard biomass from *Mycobacterium tuberculosis* and *M. leprae* [35] was processed. Long-chain compounds were extracted as described previously [26], [34] and the extract was treated with pentafluorobenzyl bromide, under phase-transfer conditions [26], [32]–[34], to convert acidic components into pentafluorobenzyl (PFB) esters. Subsequent separation on an Alltech 209250 (500 mg) normal phase silica gel cartridge gave a fraction possibly containing mycolic acid (MA) PFB esters, distinct from other lipid components [26], [32]–[34].

The MA PFB esters were reacted with pyrenebutyric acid (PBA) to produce PBA-PFB MA derivatives, which were purified on an Alltech 205250 (500 mg) C_18_ reverse phase cartridge and the PBA-PFB mycolates were analysed by sequential reverse and normal phase HPLC, all as described previously [26], [32], [34].

### Stable Isotope Analysis

In order to investigate if the different strains of leprosy could be explained by different origins of the individuals, strontium (Sr) isotopes from tooth enamel and carbon and nitrogen isotopes from bone collagen in three individuals from the Winchester leprosy Hospital were analysed for their isotope composition.

#### Sampling

Molar enamel from individuals Sk2, Sk7 and Sk8 was sampled for Sr isotopic analysis to assess if they were local to the site during adolescence. The Sr isotope composition of enamel represents the Sr isotope composition of the area where a person’s diet was being sourced during mineralisation of that tissue. A comparison of individuals’ Sr isotope ratios with proxies for the “local’ isotopic range (e.g. from plants, soils, fauna) can be used to identify immigrants to a site.

Samples of rib and femur bones from the same individuals were taken for C and N isotopic analyses to assess if there were major dietary shifts in the last decade of life, which might indicate a geographic origin away from Winchester.

#### Strontium (Sr) isotopic analysis

Sr isotopes were analysed following the method outlined [36]. The enamel surface of an intact molar was first cleaned using a dental burr and hand drill. A wedge of enamel and dentine (c. 0.5 mm wide, 1 mm deep) representing the complete growth axis of the enamel was removed using a flexible diamond impregnated dental disc. Any dentine adhering to the enamel section was then removed using a dental burr and the remaining enamel sample cleaned in an ultrasonic bath. The whole enamel section was dissolved in 1 ml 7N HNO_3_. Any detritus was removed by centrifuging and the supernatant was dried and re-dissolved in 3N HNO_3_. An aliquot of this solution was removed, representing 3 mg of solid enamel (containing approximately 100–300 ng of Sr), and made up to 0.5 ml 3N HNO_3_ to be loaded onto ion exchange columns.

The Sr was separated using standard ion exchange chromatography using 70µl of Eichrom Sr Spec resin (50–100 µm particle size) [37]. Samples were loaded in to 0.5 ml 3N HNO_3_ and washed with 4 ml 3N HNO_3_. Strontium was eluted in 1.5 ml MilliQ water (18.2 MΩ). The elutant was dried down and loaded using 1 µl 10% HNO_3_ onto rhenium filaments preconditioned with 1 µl TaCl_5_ solution and 1 µl 10% H_3_PO_4_. Isotope ratios were measured on a Thermo-Finnigan Triton Thermal Ionization Mass Spectrometer. The data is corrected for mass fractionation using a ^86^Sr/^88^Sr value of 0.1194 and an exponential mass fractionation law. ^87^Rb is subtracted using the measurement of ^85^Rb and a ^85^Rb/^87^Rb value of 2.59265 [38]. Data is corrected to NIST SRM-987 using a value of 0.710248 [39]. The typical 2σ precision for ^87^Sr/^86^Sr achieved for a tooth sample using this method is ±0.00001.

The local range for Sr isotopes was assessed from the geology. The local geology around Winchester is dominated by the Cretaceous chalk on the western edge of the South Downs [40]. Mapping of bio-available Sr in the region was completed by Evans and colleagues [41], [42]. Their work suggests that the local ^87^Sr/^86^Sr signal should be between 0.7072 (value of the Cretaceous chalk) and 0.7092 (rain water). Further to this, following the work of Bentley and co-workers [43], more detailed information on the local Sr isotope signal was obtained by analysing Sr isotopes in dentine of one individual and three rodent teeth recovered from the archaeological site.

#### Carbon and Nitrogen isotopic analysis

Collagen was extracted from bone samples using a procedure adapted from Richards and Hedges [44]. Roughly 300 mg of cleaned then cut or drilled bone was first demineralised in a 1.0 M solution of HCl at room temperature for three days. The samples were then filtered using an Ezee filter, and the acid was discarded. A solution of pH3 HCl was added to the test tubes, and the samples were gelatinized at 70°C for 48 h. The samples were filtered with an Ezee filter once more, and the remaining collagen freeze-dried for 2–3 days. Aliquots of 1–2 mg of collagen were weighed into tin capsules and sent for analysis. Isotopic analyses were carried out according to the method outlined [45]. Samples were combusted on a Carlo Erba 1108 elemental analyser system coupled to a Sercon Geo-20/20 gas source mass spectrometer operating in continuous flow mode. Isotopic values as well as elemental abundances and carbon to nitrogen ratios were calibrated against an in-house alanine standard which is traceable back to the Vienna Pee Dee Belemnite (VPDB) international standard. Further aliquots of the alanine standard were used to monitor and correct for instrumental drift. Where sufficient collagen was recovered from the archaeological bone, samples were run in duplicate. Stable isotopic results are reported in delta notation relative to VPDB for carbon and AIR for nitrogen. Alanine standards run as unknowns show the typical precision to be 0.1‰ for both δ^15^N and δ^13^C.

## Results

### Osteology

Table 2 summarises the results of the osteological analysis of the eleven individuals from whom aDNA samples were extracted. Of these eleven individuals, nine showed skeletal signs of multibacillary leprosy and this evidence is outlined in more detail below. Sk1 and Sk12 (Table 2) showed no pathology indicative of leprosy and were selected as negative controls. For a fuller description and analysis in context see Roffey and Tucker [15]. Samples from Sk8 and Sk9 were the subjects of radiocarbon dating.

**Figure 2:**
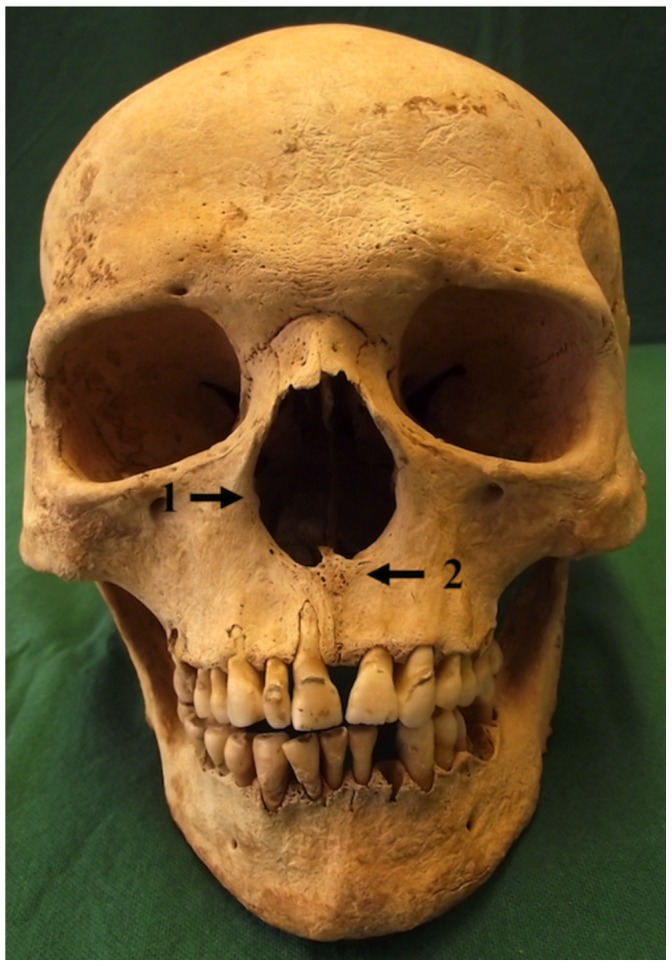
Burial Sk2, showing rounding of the margins of the nasal aperture (1) and resorption of the anterior nasal spine (2).

Sk2– an old middle adult male with evidence for circumferential wasting of the shafts of the left metatarsals and proximal foot phalanges, and porosity and loss of bone on the distal end of the distal phalanx for the left first metatarsal (the right foot was largely absent through truncation). There was also porosity of the anterior alveolar margin of the mandible and of the palate, as well as rounding, thickening and porosity of the margins of the nasal aperture, loss of the nasal spine, and porosity of the lacrimals, vomer and turbinates (Figure 2).

Sk7– a young middle adult male with evidence for porosity of the distal ends of three distal hand phalanges, circumferential wasting of the shafts of the proximal foot phalanges and porosity of the distal foot phalanges. There was also evidence for new bone on the tarsals and metatarsals, septic arthritis and loss of the arch of the left foot, and extensive and diffuse compact bone on the shafts of the tibiae and fibulae. There had also been a fracture of the distal shaft of the right fibula and fractures of the shafts of the left second and third metatarsals that were well remodelled. It is possible that these fractures are related to long-standing nerve damage and subsequent loss of full motor function. The individual also demonstrated rounding and porosity of the margins of the nasal aperture, destruction of the nasal spine, and porosity and destruction of the vomer and nasal turbinates.

**Figure 3:**
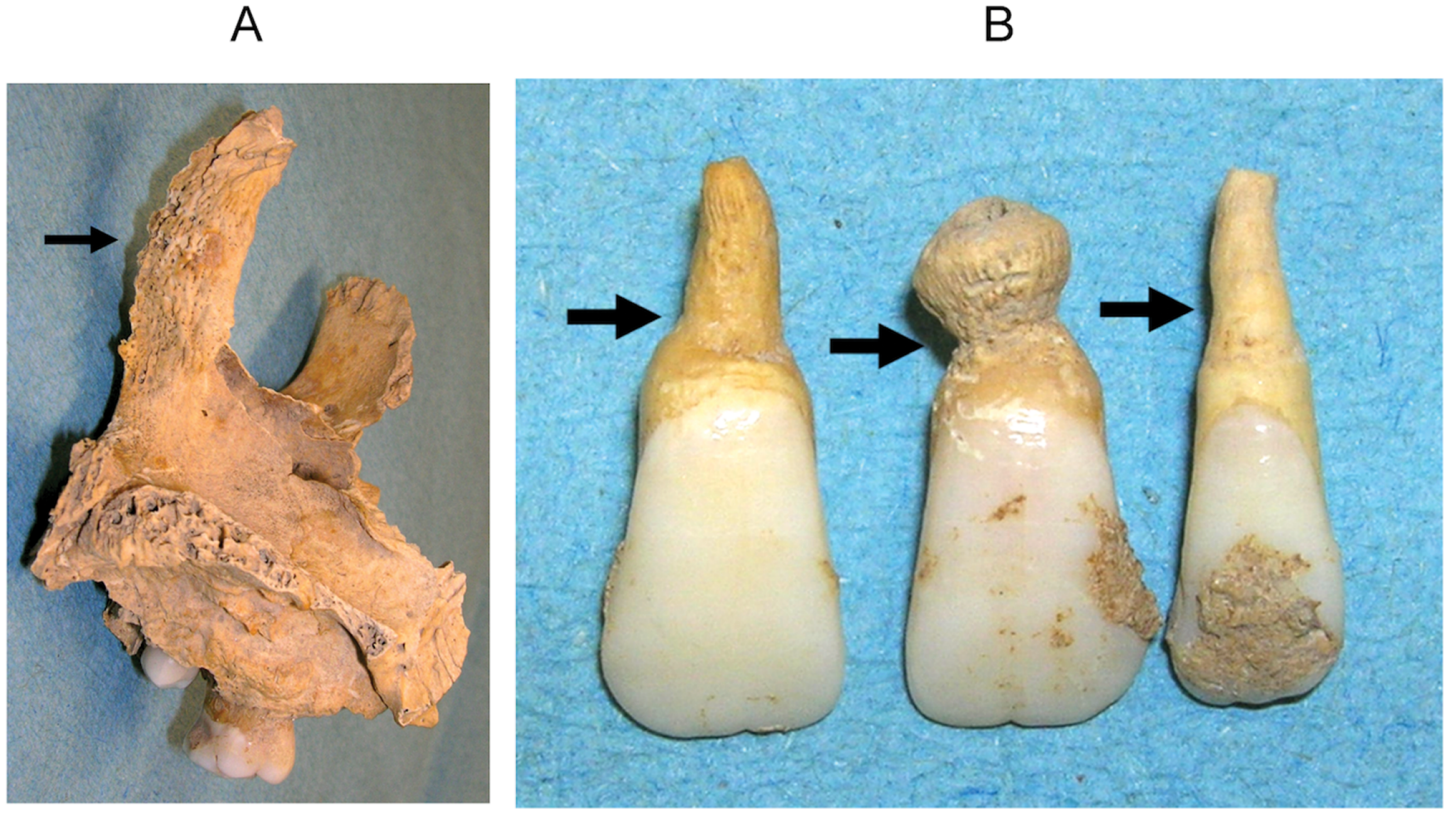
Osteological lesions in Sk8. **A.** Thickening and porosity of the internal surface of the nasal aperure. **B.** Constriction and shortening of the roots (*leprogenic odontodysplasia,* see arrows) of the maxillary incisors due to active infection with *M.leprae* during dental development.

Sk8– an older child of 9–11 years (the aDNA evidence identified this individual as male) with evidence for compact bone on the medial surface of the calcanei, porous woven bone on the shafts of the tibiae and fibulae and abnormal porosity of the alveolar bone of the mandible. There was also evidence for thickening and new bone on the internal surface of the margins of the nasal aperture (Figure 3A) and concentric restriction and reduced development of the roots of the maxillary incisors (*leprogenic odontodysplasia*, see Figure 3B). This feature was first described by Danielsen [46] in a child from the medieval leprosy cemetery at Naestved, Denmark, and is assumed to result from a disruption of dentine deposition through infiltration of the leprosy bacterium into the maxillary alveolar bone during dental development. No cases with this feature were recorded amongst the large sample of burials from Chichester and it has only been very rarely reported in the archaeological literature, with four other cases described from Denmark [46], [47], and a single case from Sigtuna, Sweden [48]. Its presence amongst the sample from Winchester is the first time the condition has been reported from outside Scandinavia, and suggests that the individual was already showing signs of active infection with the leprosy bacterium from around the age of five years. Apart from these dental changes, the other skeletal evidence for leprosy in this individual was very similar to that seen in the adult individuals with early stage leprosy. Radiocarbon C14 dating of this individual provided a calibrated date of 1010AD–1160AD (95.4% probability, lab code WK28629), suggesting the burial ground was already in use during the early-mid 12^th^ century [14], [15].

Sk9– a young middle adult male with evidence for circumferential wasting of the hand and foot phalanges with destruction of the metaphyses and joint surfaces of the foot phalanges, new bone on the tarsals and metatarsals, and diffuse compact and porous woven bone on the shafts of the tibiae and fibulae. There was no evidence for any of the features associated with the rhino-maxillary syndrome in this individual. Radiocarbon dating was undertaken on two samples. The first yielded a calibrated date of AD 980–1160 (95.4% probability, lab code WK28630). A further sample from this individual (lab code WK27734) corroborated these findings in presenting a calibrated date of AD 890–1040 (95.4% probability and 90.3% within AD 940–1040) with a clear spike in the area of AD 970–1030 [15].

Sk14– an adolescent, possible male, with evidence for circumferential wasting of the foot phalanges, porosity and loss of bone of the distal ends of the distal foot phalanges, porosity of the shafts of the metatarsals and porous woven and compact bone on the shafts of the fibulae. There was also evidence for porosity and thinning of the palate, rounding of the margins of the nasal aperture and thinning of the nasal bones.

Sk15– a young adult male with evidence for slight loss of bone on the distal end of the distal hand and foot phalanges, circumferential wasting of the foot phalanges, new bone on the tarsals and porous woven bone on the shafts of the left metatarsals and compact bone on the distal shafts of the tibiae with expansion of the distal metaphyses. There was also evidence for very slight porosity of the margins of the nasal aperture. The individual also demonstrated destructive lesions of the bodies of the eleventh and twelfth thoracic vertebrae with a sclerotic appearance to the exposed trabeculae and reactive bone on the anterior surfaces of the bodies. The appearance of these lesions would largely correspond to that expected in spinal tuberculosis [3], although the reactive bone on the anterior of the vertebral bodies, and the absence of any evidence for collapse of the bodies, despite the loss of much of the internal structure of the body, would not be typical. A possible differential diagnosis for the vertebral lesions would be brucellosis, a highly infectious disease acquired through contact with infected animals and usually transmitted to humans via infected meat and milk. It manifests as a severe respiratory illness and also causes destruction of joints and vertebrae, hence the difficulties in distinguishing its effects on the skeleton from those of tuberculosis. The reactive bone on the vertebrae and the absence of any collapse of the bodies in SK15 would be more typical of brucellosis, although its appearance in the vertebrae is usually multi-focal, whereas those in this individual are restricted to the eleventh and twelfth thoracic vertebrae [49], [50].

Sk18– an adolescent, possibly male, with evidence for circumferential wasting of the hand phalanges, porosity, cortical and trabecular atrophy of the tarsals, metatarsals and foot phalanges with blade-like destructive remodelling of the metatarsals and shell-like expansion of the foot phalanges, porosity and new bone on the shafts of the tibiae and fibulae with thickening and disorganisation of the cortical bone. There was also evidence for porosity of the alveolar borders of the maxilla and mandible, porosity of the palate, rounding of the margins of the nasal aperture and loss of the nasal spine. The individual also had a very broad and enlarged cranial vault, a flattened nasal bridge and prognathism of the maxilla. These features may be unrelated to leprosy and evidence for a congenital or developmental anomaly, however, a very similar facial appearance is seen in an adolescent individual from Naestved, Denmark, who also had evidence for rhino-maxillary leprosy [47], and may therefore be related to active infection with the leprosy bacterium during skeletal development.

**Figure 4:**
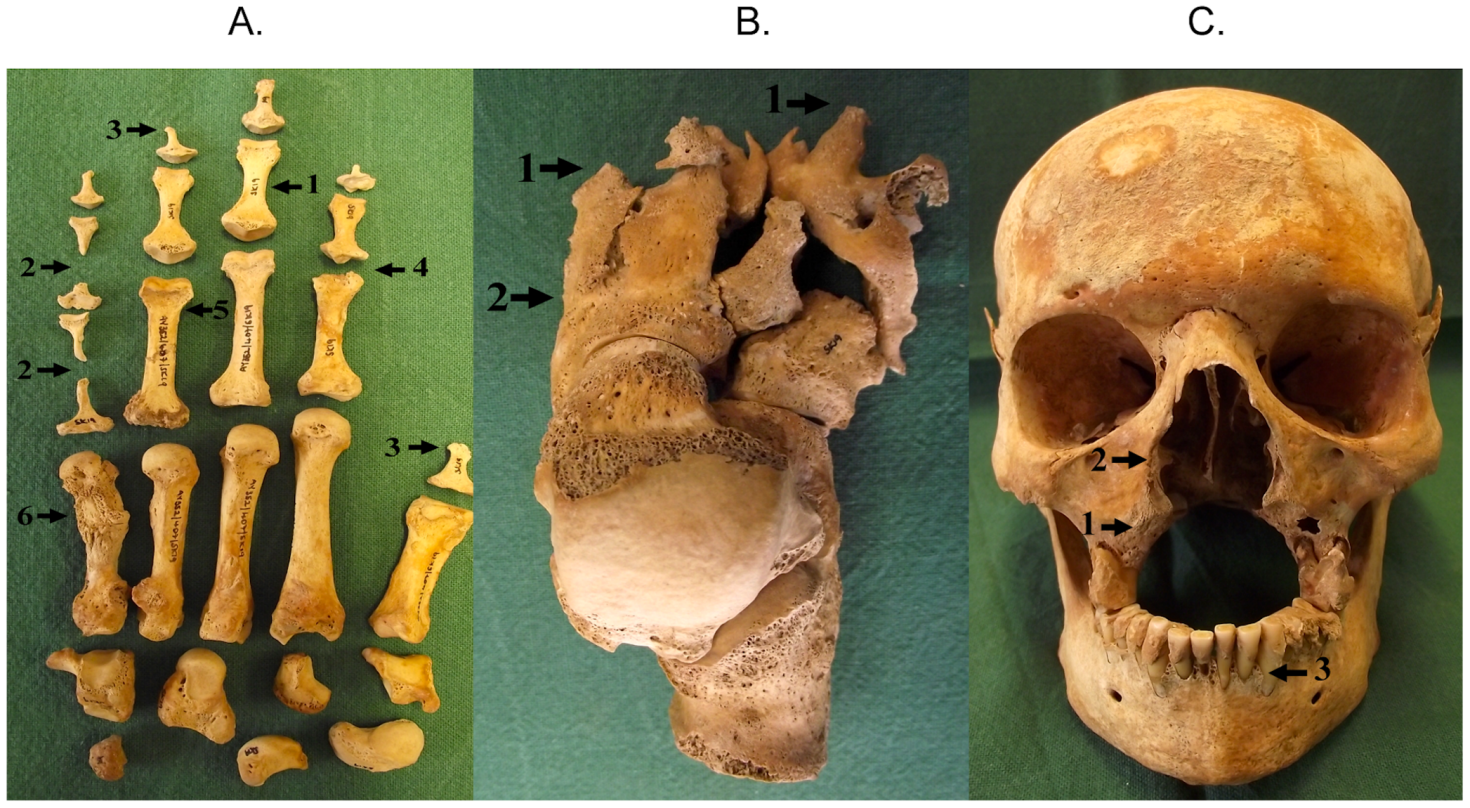
Osteological lesions in burial Sk19. **A.** Circumferential wasting of the shafts of the hand phalanges (1) (with complete resorption of the shafts of the proximal and medial phalanges of the fifth digit), (2), resorption of the ends of the digits (3), destruction of the joints (4), volar grooving of the proximal phalanges (5), and diffuse new bone on the shaft of the fifth metacarpal (6). **B.** Complete loss of the pedal phalanges, near complete destruction of the metatarsals (1) and fusion of the tarsals (2). **C.** Complete resorption of the anterior maxillary alveolar bone and palate (1), rounding of the margins of the nasal aperture (2) and porosity of the mandibular alveolar bone (3).

Sk19– a young middle adult, possibly male, with evidence for circumferential wasting of the metacarpals and hand phalanges with complete resorption of the shafts of some of the proximal and medial phalanges (Figure 4A), and loss of bone in the distal shafts of the distal phalanges, destruction of the joint surfaces between the proximal and medial phalanges and volar grooving of the proximal phalanges and new bone on the shafts of the metacarpals with diffuse bone on the shaft of the right fifth metacarpal. There was also evidence for new bone on the distal shaft of the right radius, the left femur and both tibiae and fibulae. The distal end of the left tibia and fibula were absent with tapering and flattening of the ends of the bones, cortical atrophy and ankylosis by bony bridging at the distal ends of the shaft. This seems to be consistent with a deliberate amputation through the distal ends of the bones of the lower limb and the degree of remodelling indicates this must have occurred sometime before death. The right tarsals were porous with fusion of the navicular, and first and second cuneiforms, and destruction of the joint surfaces of the cuneiforms and cuboid. The metatarsals were also fused with blade-like resorption of the majority of the shafts and there had been complete loss of the foot phalanges (Figure 4B). The grave fill was retained and sieved, with no fragments of the phalanges being recovered. There was also evidence for flattening, fusion and resorption of the nasal bones, resorption of the zygomatics, rounding and resorption of the margins of the nasal aperture, resorption of the alveolar border of the mandible and severe resorption of the maxillary alveolar process and palate, with loss of bone extending back to the first molars (Figure 4C).

Sk23– a young middle adult possible female (the aDNA sex determination identified this individual as male) with evidence for circumferential wasting of the shafts of the metatarsals and foot phalanges with complete resorption of the shafts of some of the phalanges and destruction of the distal joint surfaces of the metatarsals, resorption of the distal ends of the distal foot phalanges, volar grooving of the proximal hand phalanges, septic arthritis of the left wrist and left foot, porous woven bone on the shafts of the metatarsals and diffuse new bone on the shafts of the tibiae and fibulae. There was also evidence for fractures of the shafts of the left second and third metatarsals that may be related to loss of full motor function. The cranium was absent through truncation and therefore an assessment of any rhino-maxillary changes could not be made.

### Biomolecular Study

#### PCR

**Figure 5:**
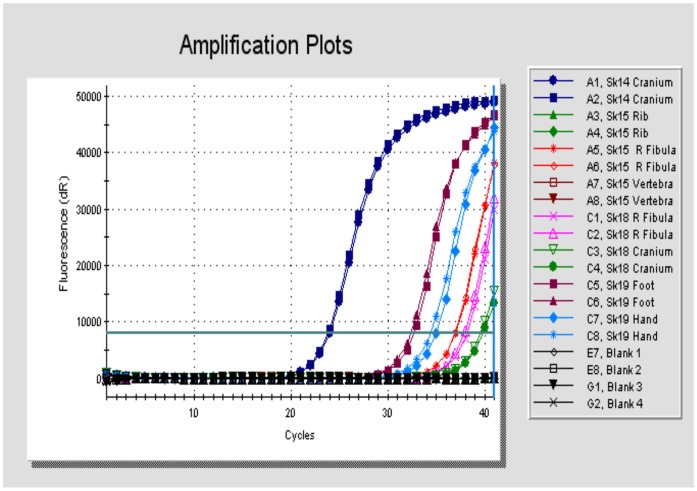
Real-time PCR amplification profiles for cases Sk14, Sk15, Sk18 and Sk19 obtained using the RLEP PCR method with the intercalating dye EVAGreen. Estimations were performed in duplicate. NTC (black symbols) = Non-template controls.

Table 4 summarises the results of screening the burials for remnant *M. leprae* DNA and also for any evidence of MTB complex DNA. Of the nine individuals showing lesions suggestive of LL, all were positive for *M. leprae* using the sensitive multiple- copy RLEP PCR. The control specimens (Sk1 and Sk12), with no osteological evidence of leprosy, were negative. Six of these nine RLEP-positive cases were also positive using the single-copy 18-kD antigen PCR. Three cases, Sk9, Sk15 and Sk18 were negative by 18-kD PCR and only weakly positive by RLEP PCR. A fourth burial, Sk23 was weakly positive using both markers. These four cases were not taken further. Cases Sk2 and Sk14 were consistently the most robust positives, followed by Sk19. These always exhibited lower Cq (quantification cycle) values, indicating amplification of product after fewer cycles than the remaining cases. The Cq is defined as the cycle number in which fluorescence first increases above a defined threshold level and is inversely related to the log of the original template copy number. Figure 5 shows typical amplification profiles of burials Sk14– Sk23 obtained using the RLEP real-time PCR. It can be seen that surviving *M. leprae* DNA, as judged by the Cq values, varied not only between burials but also between different skeletal components in the same individual. For example, extracts prepared from foot bones of Sk19 exhibited mean Cq values of 32.38 compared with 34.5 for hand bone extracts. A similar observation was made in Sk7, where Cq values were always lower in extracts prepared from nasal conchae compared with foot bones (Table 4). Dissociation curve analysis of the RLEP amplicon in these and other experiments showed melt temperatures in the correct range (90 to 92°C) and subsequent gel electrophoresis showed single products of expected size (111 bp, not shown).

**Table 4:** Real-time PCR data for *M. leprae* screening methods (RLEP & 18-kD) and for MTB complex DNA (IS*1081*).

Burial	Extract	RLEP PCR	Cq values^1^ (RLEP)	18-kD PCR	Cq values^1^ (18-kD)	IS*1081* PCR
Sk1	Palate	−	No Cq	−	No Cq	−
	Nasal conchae	−	No Cq	−	No Cq	−
Sk2	Foot phalanx	++	28.09	nd	nd	−
	Cranial fragment	++	26.5	+	32.78	−
Sk7	Foot phalanx	+	37.0	−	No Cq	−
	Nasal conchae	+	32.33	±	38.59	−
	Ossified tibial ligament	±	38.74	−	No Cq	−
Sk8	R Fibula 1 new bone	++	25.08	nd	nd	−
	R Fibula 2 next to 1	+	32.20	±	38.43	−
	L maxilla	+	29.55	+	35.68	−
	Ethmoid bone	+	36.01	−	No Cq	−
Sk9	Foot phalanx	−	No Cq	nd	nd	−
	L tibia, proximal end	±	37.47	nd	nd	−
Sk12	Nasal conchae	−	No Cq	−	No Cq	−
Sk14	Cranial fragment	++^a^	23.71	++	28.75	−
	Nasal conchae	++	27.91	+	34.22	−
	Metatarsal	++	27.11	+	33.98	−
Sk15^2^	R fibula	±	37.77	−	No Cq	−
	Vertebra (1) T11	−	No Cq	−	No Cq	−
	Vertebra (2) T11	−	No Cq	−	No Cq	−
	Rib	−	No Cq	−	No Cq	−
Sk18	R fibula	±^a^	38.46	−	No Cq	−
	Cranial fragment	±	38.86	−	No Cq	−
Sk19	R foot	±^a^	32.38	±	37.3	−
	R metacarpal (1)	+	34.5	±	38.14	−
	R metacarpal (2)	+	34.49	±	38.52	−
	Dental calculus	+	36.29	−	No Cq	−
Sk23	R 4^th^ metatarsal	±^a^	37.99	±	38.6	−
	R. fibula	±	38.4	−	No Cq	−

1 = Mean of duplicates.

2 = Osteological evidence of tuberculosis or brucellosis.

a = RLEP PCR confirmed at the second centre.

Scoring scheme for individual cases: ± = weak positive;+ = positive;++ = strong positive.

**Figure 6:**
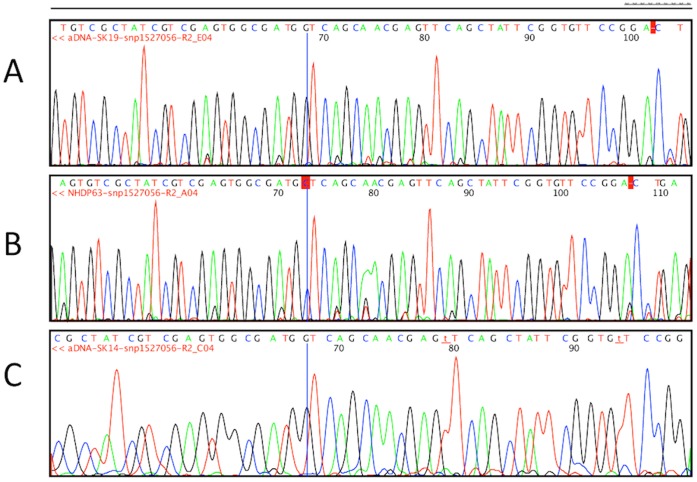
Automated DNA sequencing of the ML1527056 locus from A.Sk19. B.Reference strain NHDP63 and C.Sk14.

Five of the six burials positive for both screening tests, RLEP and 18-kD antigen, were taken forward for genotyping. These were cases Sk2, Sk7, Sk8, Sk14 and Sk19. A series of PCR methods were applied to type the isolates using phylogenetically informative loci [26], [27]. The results are shown in Table 5. Three cases were found to be type 3I. These were further sub-typed using polymorphisms identified in SNP 1,527,056 and inDel_17915 [30]. All isolates exhibited base G at SNP 1527056 (Figure 6) and only one copy of an 11 bp repeat, TTGGTGGTGTA, indicating that these isolates were consistent with 3I-1 strains. Perhaps most interestingly, the remaining two cases, Sk8 and Sk14 were found to be type 2, not previously identified in archaeological material from Britain. Additional PCRs for SNP loci 1,104,232 and 3,103,778 [30] identified these isolates as type 2F. Two burials, Sk1 and Sk12, which were sampled to act as negative controls were found to be free of any evidence of either *M. leprae* or MTB complex DNA.

**Table 5:** Summary of SNP genotyping data.

Locus	Sk2	Sk7	Sk8	Sk14	Sk19
SNP 1 14,676	C	C	C	C*	C*
SNP 2 1,642,875	T	T	T	T*	T
SNP 3 2,935,685	C	C	A	A	C*
SNP 4 413,902	G	G	G	G	nd
SNP 5 591,857	C	C	C	C	nd
SNP 6 1,133,492	T	T	T	T	T*
SNP 7 2,312,059	C	C	C	C	nd
SNP 8 7,614	T	T	C	C*	Fail
SNP 9 1,113,923	G	G	A	A	A
SNP 10 1,104,235	nd	nd	C	C*	nd
SNP 11 3,102,787	nd	nd	C	C*	nd
InDel_1791511 bp repeat	1 copy	1 copy	2 copies	2 copies*	1 copy
SNP 12 1,527,056	G	G	nd	G*	G*
**Genotype**	**3I-1**	**3I-1**	**2F**	**2F** *	**3I-1** *

* = Also replicated at centre two. nd = not determined.

#### MLVA typing

The data obtained for MLVA typing is shown in Table 6. Sequence data for the three chosen loci was obtained from four of the five cases, Sk2, Sk7, Sk8 and Sk14. Typing of Sk19 was problematical and the 21-3 locus failed to amplify. However, the remaining two loci, (AGA)20 and (GTA)9 were amplified and successfully sequenced. These revealed 14 and 7 copies respectively. The (AGA)20 locus was found to be the single most useful VNTR locus in the Winchester cases, with a total of 3 alleles found. Burials Sk2 and Sk7 exhibited 11 and 13 copies of this trinucleotide whereas the remaining cases all contained 14 copies. Both type 2F strains (Sk8 and Sk14) contained 14, 8 and 2 copies of (AGA)20, (GTA)9 and 21-3 respectively and so could not be differentiated by MLVA typing with the applied methods. Indeed, the 21-3 locus was identical in all instances where a product was obtained, showing two copies of the repeat motif.

**Table 6:** MLVA typing of *M. leprae* isolates recovered from Winchester leprosy cases.

Burial	(AGA)20 (TTC)	(GTA)9	ML0058 (21-3)
Sk2	11	8	2
Sk7	13	8	2
Sk8	14	8	2
Sk14	14	8	2
Sk19	14	7	Fail

#### Other PCR tests applied

Repeated testing of Sk15, which showed convincing skeletal evidence of Pott’s disease in several thoracic vertebrae, was MTB DNA negative. As brucellosis was one of the likely differential diagnoses for the lytic vertebral lesions, the extracts from Sk15 were also tested for *Brucella spp*. bacteria using a real-time PCR method for the multi-copy IS*711* element [51]. This assay was also negative.

Sex assessment with amelogenin PCR was successful in 9 of the 10 cases tested (Table 7). All individuals were found to be male, including the child skeleton Sk8, where osteological determination of sex was precluded. Sk15 was tested but no PCR products were seen, indicating poor DNA preservation and offering one possible explanation for failing to detect MTB complex or *Brucella* DNA in thoracic vertebrae tested with IS*1081* and IS*711* PCR respectively. The only discrepancy between biomolecular and morphological analyses was Sk23, which was recorded as possibly female by skeletal indices but was found to be male by amelogenin PCR.

**Table 7:** Sex of leprosy cases determined by osteological and biomolecular methods.

Burial	Sex by osteological analysis	Amelogenin PCR amplicons (bp)	Sex by amelogenin PCR
Sk1	Male	105	Male
Sk2	Male	105 & 290	Male
Sk7	Male	105 & 290	Male
Sk8	nd (older child)	105	Male
Sk9	Male	nd	nd
Sk12	Male	105	Male
Sk14	?Male	105 & 290	Male
Sk15	Male	PCR negative	nd
Sk18	?Male	105 & 290	Male
Sk19	?Male	105	Male
Sk23	?Female	105 & 290	Male

nd = not determined.

#### Replication of key findings at centre two

Samples Sk14 and Sk19 were successfully genotyped as SNP subtype 2F and 3I-1 respectively. In two other samples, Sk18 and Sk23, *M. leprae* DNA could be detected only upon targeting the multicopy repetitive sequence RLEP (111 bp amplicon). These observations match the findings of centre one.

#### Sequencing

We did not find any deviation at polymorphic loci from the typing schemes already proposed by Monot and colleagues [27] or Truman and co-workers [30] for 3I strains.

### Lipid Analyses

**Figure 7:**
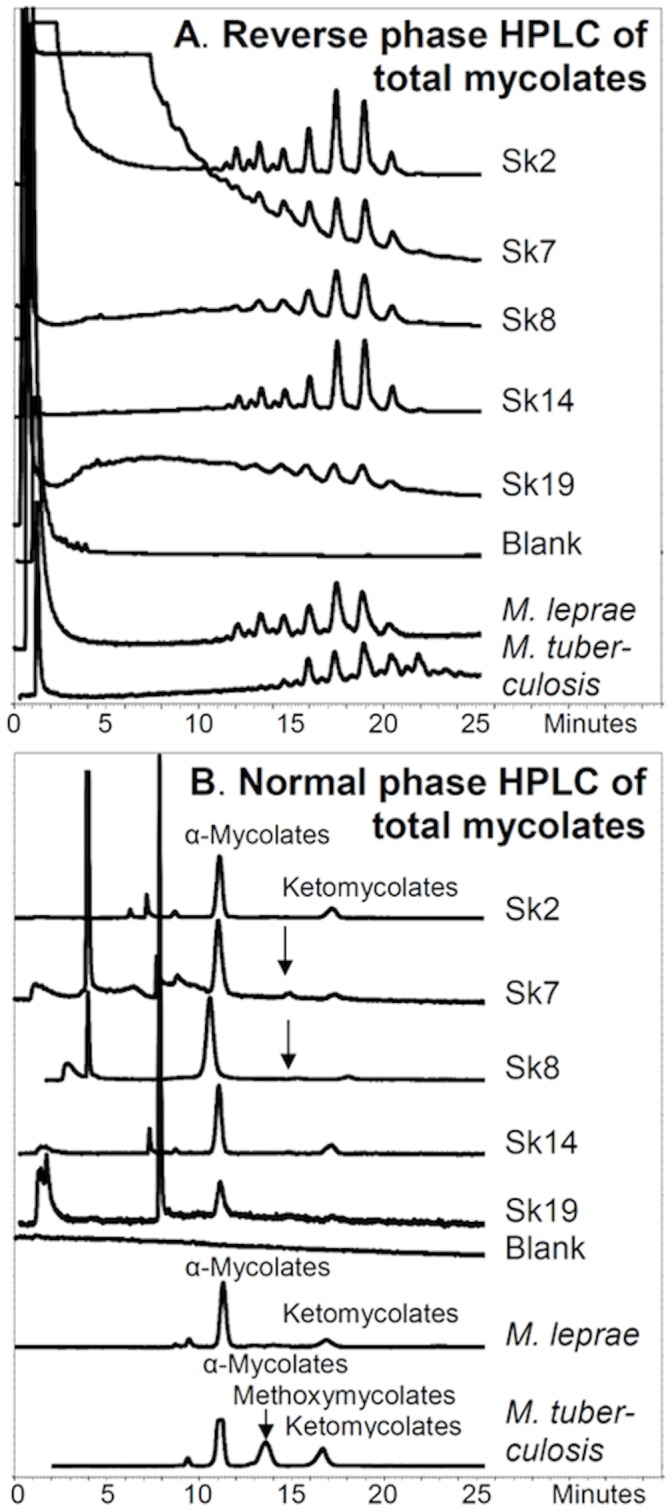
Fluorescence HPLC of PBA-PFB derivatives of total mycolic acids from Winchester leprosy cases. **A.** Reverse phase HPLC of total mycolates. **B.** Normal phase HPLC of total mycolates collected from the reverse phase separation. Vertical arrows show the position of collected material, which gave a weak unknown profile on subsequent reverse phase HPLC.

Reverse phase HPLC of the PBA-PFB mycolate fractions indicated the presence of long-chain mycolates in all samples, but the profile for Sk19 was very weak and inconclusive (Figure 7A). The profiles for Sk2 and Sk14 were very strong, correlating well with the standard *M. leprae* profile, as did the weaker profiles for Sk7 and Sk8. The total standard *M. leprae* mycolate profile is distinguished from that of standard *M. tuberculosis* (Figure 7A) by the presence of components eluting between 10 and 15 minutes; as shown in the illustration, these components correspond to the smaller ketomycolates characteristic of *M. leprae*. The total mycolate profiles (Figure 7A), in themselves, are already indicative, therefore, of leprosy in the samples studied, with reservations regarding Sk19.

**Figure 8:**
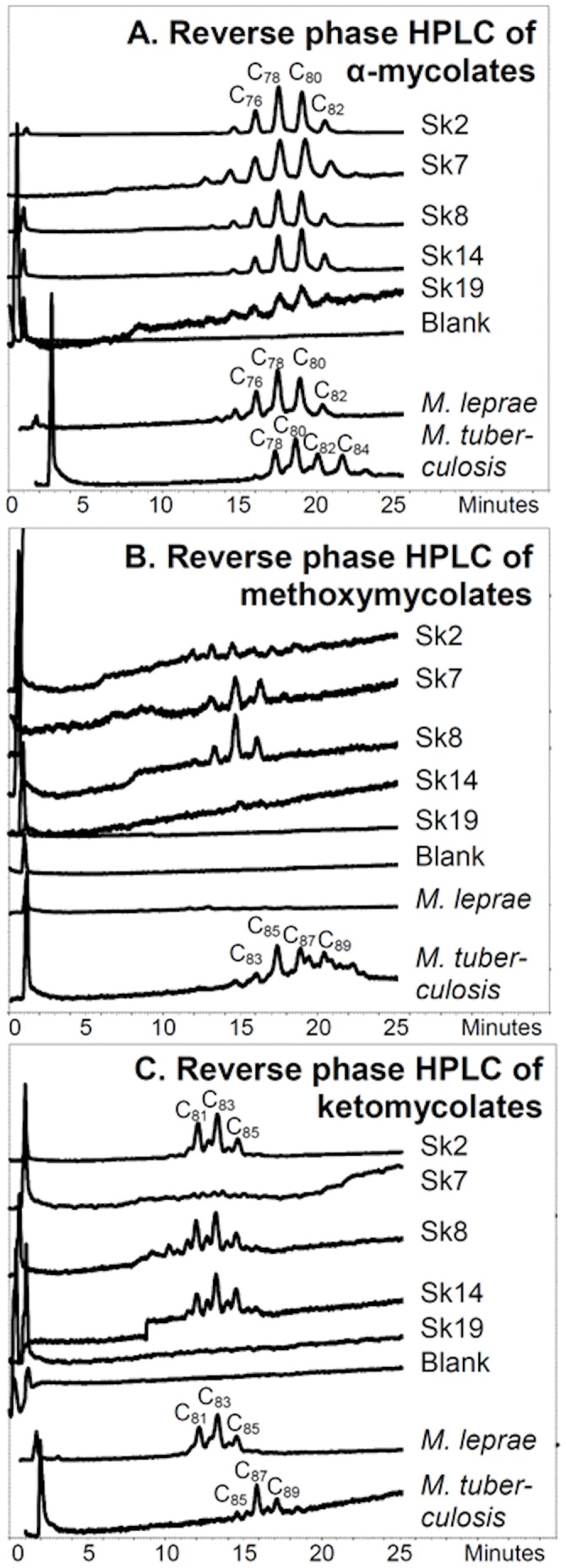
Reverse phase fluorescence HPLC of PBA-PFB derivatives of mycolate classes collected from the normal phase separation. **A.** α-Mycolate fraction. **B.** Methoxymycolate fraction. **C.** Ketomycolate fraction. Peaks are labelled to indicate the numbers of carbons in the parent underivatised mycolic acids; for example, C_80_ is a free mycolic acid with 80 carbons overall.

The total components in the region corresponding to mycolates, from the reverse phase HPLC analyses (Figure 7A), were collected and analysed by normal phase HPLC (Figure 7B). The α-mycolates were recorded in all cases, accompanied by small proportions of ketomycolates, except for Sk19. No substantive peaks for methoxymycolates were observed, but for Sk7 and Sk8, material collected from the region associated with methoxymycolates (see arrows in Figure 7B) did give weak unknown profiles on subsequent reverse phase HPLC (Figure 8B). The balance of evidence in the normal phase total mycolate profiles in Figure 7B favours leprosy over tuberculosis. The total mycolates from *M. tuberculosis* are characterised by the presence of substantial proportions of methoxymycolates, but this mycolate type is not produced by *M. leprae* [35].

Reverse phase HPLC profiles of the collected fractions from the normal phase HPLC separations (Figure 7B) are shown in Figure 8. The profiles of the α-mycolate fractions (Figure 8A) all showed correspondence with the *M. leprae* standard, including the very weak trace from Sk19. Excellent ketomycolate profiles were recorded for Sk2, Sk8 and Sk14, correlating with that for standard *M. leprae* (Figure 8C). In order to test for the possibility of any tuberculosis infection, characterised by the presence of methoxymycolates, the intermediate material collected between the α-mycolate and ketomycolate fractions (Figure 7B) was analysed by reverse phase HPLC (Figure 8B). Weak profiles were observed for SK7 and SK8, with an even weaker suspicion in Sk2. However, the profiles did not overlap with authentic *M. tuberculosis* methoxymycolates, so there is no positive evidence for tuberculosis in these samples taken from the area of skeletal leprotic lesions.

### Stable Isotope Analysis

**Figure 9:**
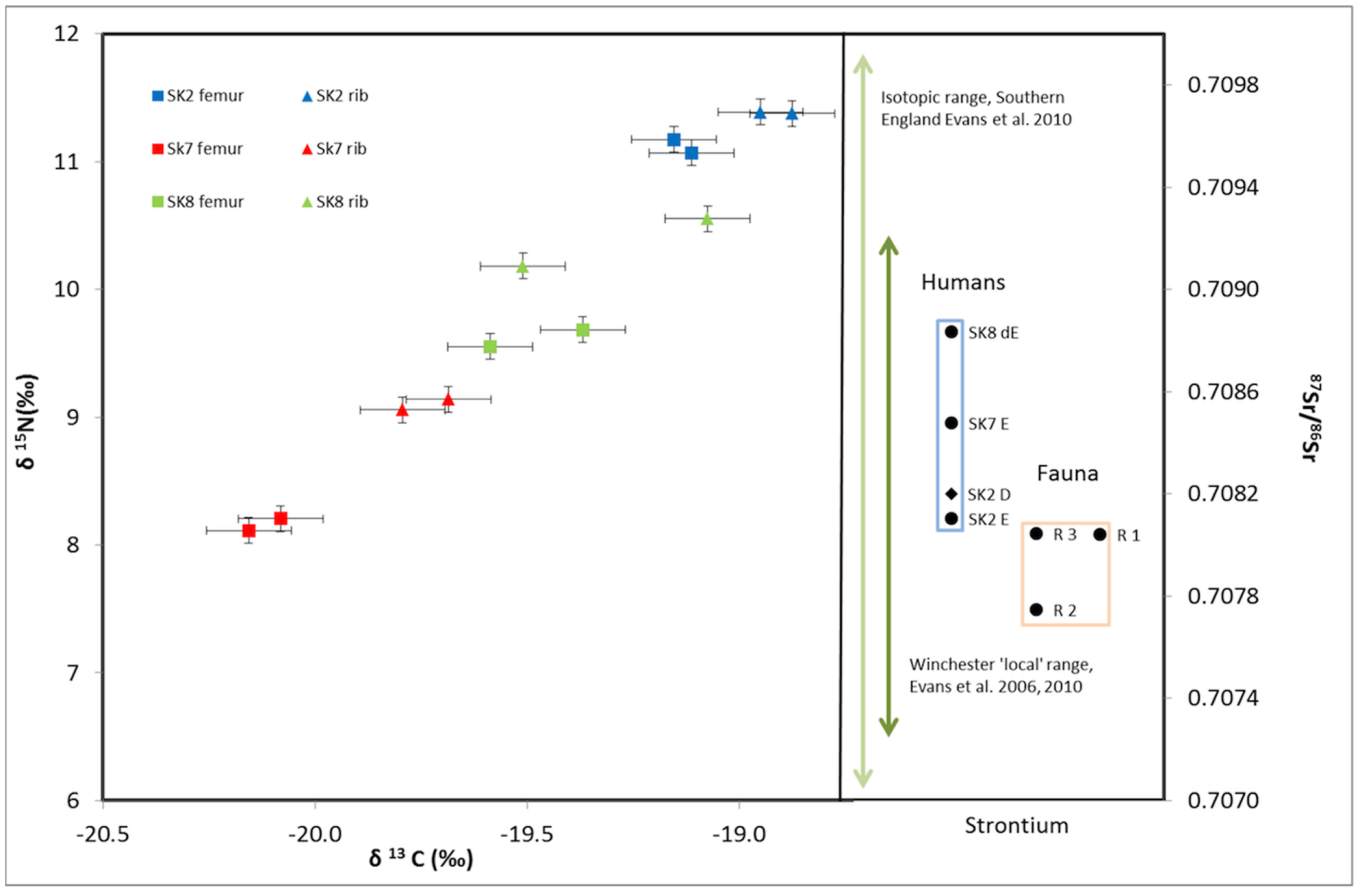
Dietary isotopes (d^13^C and d^15^N) and ^87^Sr/^86^Sr values for three leprous individuals. Measurements for the dietary isotopes are in duplicate and on both femurs and ribs representing bones with slow (femur) and faster (rib) collagen turnover rates [54]. d^13^C and d^15^N values are consistent with the expected diet of a northern European terrestrial C3 diet, with perhaps a small marine component. Elevated d^13^C and d^15^N of the ribs over the femur samples for each individual suggests increased marine or freshwater resource consumption later in life. The Sr isotope analyses are on enamel (E) and also one on dentine (D) to represent the diagenetic Sr signal. Three faunal whole tooth Sr isotopic values (R1-R3) are included to estimate the ‘local’ Sr range as are published isotopic ranges for Southern England and the Winchester region. The three human enamel samples fall within the isotopic range for the Winchester region and are therefore consistent with a Southern England childhood origin for these individuals.

Strontium isotope ratios for the three individuals from the hospital are consistent with having spent their adolescent years in the region surrounding Winchester (Figure 9; Table 8). Sk2 and Sk7 have Sr isotope ratios within the defined local range [41], [42]. Sk8 does have an ^87^Sr/^86^Sr value, which is slightly elevated however this difference is not significant as it is still below the upper limit of the local range. Other studies which have combined Sr and oxygen studies in Winchester have identified ‘local’ people with similar ^87^Sr/^86^Sr [40]. Furthermore, the dentine result, which is expected to be diagenetically altered to look similar to the local soil Sr values, falls within the isotopic range of the three individuals. Finally, while the small rodent teeth are marginally less radiogenic than the humans, the difference is well within the variation expected from a local population [42].

**Table 8:** Sr, C and N isotopes for humans and fauna.

Sample	^87^Sr/^86^Sr	δ^13^C rib (‰)	δ^15^N rib (‰)	δ^13^C femur(‰)	δ^15^N femur(‰)
Sk2 E	0.708104± (7)	−18.91	11.38	−19.13	11.12
Sk2 D	0.708201± (6)	n/t	n/t	n/t	n/t
Sk7 E	0.708478± (7)	−19.74	9.10	−20.12	8.16
Sk8 E	0.708833± (10)	−19.29	10.37	−19.48	9.62
Rodent 1	0.708040± (6)	n/t	n/t	n/t	n/t
Rodent 2	0.707748± (7)	n/t	n/t	n/t	n/t
Rodent 3	0.708045± (7)	n/t	n/t	n/t	n/t

For Sr, E denotes enamel, D dentine. Rodent teeth are analysed as whole teeth. δ^13^C and δ^15^N values are the mean of two samples, typical precision ±0.1‰. n/t = not tested.

C and N isotopes were measured in both rib and femur bone collagen. Trabecular bone turns over 3–10 times faster than cortical bone (ICRP 1975) [52], so the isotopes from the ribs will represent the more recent diet than from the femur samples. If an individual were to move from a region where a C_4_ diet was prevalent to one where C_3_ plants dominate, within the last decade of their life, we would predict differences in the δ^13^C between the rib and femur samples.

Femur carbon isotope results for all three individuals are consistent with the expected diet of a northern European consuming a terrestrial C_3_ diet (Figure 9; Table 8). Carbon isotope values for the Winchester area are expected to fall in the range of terrestrial C_3_ diet (δ^13^C, −20 to −18‰) as there were no economically important C_4_ plants in North-western Europe until recent times [53], [54]. This precludes the possibility of a significant C_4_ input into the diet, which might be typical of an individual with a Near Eastern, or Eastern European origin. Femur nitrogen isotopes for Sk7 and Sk8 fall within the expected range of humans with intermediate trophic level between herbivore and carnivore. Femur nitrogen for Sk2, who had type 3I leprosy are slightly elevated above the expected range, this may indicate a small component of marine protein in the diet. This in turn is consistent with his marginally more positive carbon values.

The three individuals show enrichment in δ^13^C and δ^15^N in ribs relative to femurs; all three individuals had a shift in their diet in the final years of their lives. This can be interpreted as being the result of increased consumption of marine resources, perhaps once the patients with leprosy entered the hospital. Numerous authors have used stable isotopes to highlight the importance of fish in the medieval period [55], [56], where monastic communities relied on marine and freshwater fish in order to supplement diets due to the exclusion of meat eating on certain days and during fasts [57]. Although the leprosy patients in the hospital were not part of the monastic order, they may have had a similar diet and dietary restrictions imposed on them once they became patients.

If all individuals at the hospital had the same diet then it would be expected that their rib collagen and then femur collagen would converge on a single isotopic value. However, the ribs and femurs differ between the individuals and show a consistent pattern of enrichment in rib δ^13^C and δ^15^N relative to femur δ^13^C and δ^15^N. This suggests that the ribs have only partially equilibrated with the hospital diet and may imply that the individuals died within a few years of entering the hospital.

## Discussion

The site of St Mary Magdalen leprosy Hospital, Winchester, has been the focus of excavation by the University of Winchester since 2008 [13], [58], [59]. The excavations (directed by SR and PM) have so far revealed evidence for a number of the medieval buildings associated with the hospital, as well as a relatively large group of burials (56 individuals have so far been excavated from the hospital as a whole [15]. The earliest phases of occupation on the site included a small masonry structure with a series of post-pits, post-holes, linear features, and an associated cemetery. Most of these features underlay the later masonry phases of the mid-12th century hospital and probably represented a masonry chapel with associated timber structures and cemetery. Scientific dating and diagnostic material culture suggest that this early phase dated to the immediate decades after the Norman Conquest between AD 1070 and 1100 and it may therefore be a contemporary to the first documented hospital founded by Archbishop Lanfranc at Canterbury in the 1080s [14], [15], [60]. Of the burials from the early, northern cemetery, 38 were excavated and comprised anthropomorphic graves with head niches toward the west. Selected cases from the 38 burials from this ‘northern’ cemetery form the basis for this analysis. Of the 38 burials, 33 showed indications of leprosy (86.8%) as well as other diseases, and they also included a medieval pilgrim (Sk27). One individual (Sk19), presented evidence for an amputated lower left leg and related medical care. Two of the burials were sampled for C14 dating. One of these burials (Sk9), which also presented evidence for leprosy, was of some significance in that it presented a possibility of a pre-Norman Conquest burial (10^th^–12^th^ century, see Results). A further sample from that same area (Sk8), however, provided a broader Post-Conquest date to one of the stratigraphically latest additions to the cemetery and suggests the burial ground was in use during the early-mid 12th century. This would be consistent with the associated physical material evidence and argue for an early Norman date (mid-late 11th century) for the cemetery. The hospital was likely refounded during the mid-late 12th century when the timber buildings (and cellared feature) were replaced by a large masonry hall and the chapel was substantially rebuilt. The northern cemetery was probably decommissioned at this time as the later buildings sealed much of it.

The skeletal manifestations of leprosy in archaeological skeletons were first observed and described by Møller-Christensen [61]–[64] who based his research on the examination of skeletal remains associated with medieval leprosy hospitals in Denmark. He was the first to describe the distinctive changes of the facial skeleton, for which he used the term *facies leprosa*, and which encompassed the rounding and enlargement of the margins of the nasal aperture, the destruction of the nasal spine and the resorption of the maxillary alveolar bone. The appearance of destructive remodelling of bone in these areas of the cranium are now referred to as rhino-maxillary syndrome and are an important diagnostic feature in skeletal leprosy [23].

Further work on leprosy in skeletal remains was undertaken by Jobs Andersen, in collaboration with Keith Manchester and others, and was focused on the material from the Danish hospitals as well as the large sample of skeletons excavated from the medieval hospital site of St. James and St. Mary Magdalene, Chichester [65]–[69].

Study of the *M. leprae* genome itself, shows a significant downsizing of genetic material and loss of metabolic repertoire in the distant past [70]. This reductive evolution suggests specialisation and a change in lifestyle from free-living mycobacteria to obligate pathogen at some point in antiquity, probably after an evolutionary bottleneck, which likely affected a number of mycobacterial species to variable degrees [71]. It is apparent that genome downsizing long predates the global dissemination of the pathogen and its interaction with different human populations.

It is likely that recombination events between interspersed repetitive sequences such as RLEP (37 copies), REPLEP (15 copies), LEPREP (8 copies) and LEPRPT (5 copies), was the mechanism driving gene deletion, translocation and inversions, which are the trademark of the leprosy genome. In the present bioarchaeological study of early medieval cases, we have used one of these repetitive sequences (RLEP) as a sensitive screening real-time PCR method for evidence of remnant DNA from isolates of *M. leprae* (Figure 5).

An examination of informative single nucleotide polymorphisms (SNPs) in strains recovered from regions where the disease is still endemic has shown that present-day cases of leprosy are derived from a clone which probably originated in East Africa and spread with successive human movements around the world [4]. Four main genotype profiles have been identified and recently, recognition of additional phylogenetically informative SNPs has allowed sub-typing of the four main groups into a total of 16 subtypes [27]. The SNP type 4 subtypes (N, O and P) do not show nucleotide base changes but are defined on the basis of variation in insertion and deletions (InDels) and homopolymeric tracts (HPT).

The cases of leprosy examined in the current study are therefore of interest in terms of the aetiology, history and global dissemination of the disease. The skeletons recovered from the site of the leprosy hospital showed excellent morphological preservation and this was reflected in the ability to recover and amplify evidence of *M. leprae* DNA from all 9 cases examined. The fact that the earlier northern cemetery was covered by later medieval buildings until into the 18th century may be one reason for this degree of preservation. The survival of mycobacterial DNA was such that five of the nine burials yielded valuable SNP and MLVA data, permitting the leprosy isolates to be placed within the evolutionary scheme for this pathogen and identified as unique isolates.

Consistent with expected distribution of mycobacterial DNA with skeletal lesions, the rhino-maxillary area showed lower Cq values compared with other sites such as dental calculus, or fibulae, probably reflecting a higher mycobacterial burden at the time of death. The more robust cases all provided evidence for multiple regions on the *M. leprae* genome and the genotyping data make phylogenetic sense, which is one of the more stringent tests of authenticity. Four cases were studied at the second centre; two of these in detail and the SNP profiles exactly matched the findings at the first centre. Additional confirmation for presence of *M. leprae* was provided by mycolic acid analysis.

Mycolic acid profiles from standard *M. leprae* [26], [35] are distinguishable from those of standard *M. tuberculosis* [32], [34]. In particular, *M. leprae* lacks methoxymycolates and the α-mycolates and ketomycolates are significantly smaller in range (Figures 7 and 8). The good α-mycolate and ketomycolate profiles, recorded for extracts of Sk2, Sk8 and Sk14 (Figures 8A and 8C), are firm evidence for the presence of leprosy. Leprosy is also highly probable in Sk7, as evidenced by an excellent α-mycolate profile; even the very weak α-mycolate profile from Sk19 (Figure 8A) is suggestive of leprosy. As noted above, methoxymycolates are not produced by standard *M. leprae* (Figures 7B and 8B), but fractions collected from the methoxymycolate region of the normal phase HPLC analyses (Figure 7B) gave clear weak reverse phase profiles, particularly for extracts of Sk7 and Sk8 (Figure 8B). The trace components recorded in Figure 8B do not correspond to the methoxymycolates from *M. tuberculosis*, but the possibility that they represent degradative modification products of the α-mycolates or even ketomycolates must be considered. The presence of these unusual components must be monitored in future studies to assess whether they have any particular significance for the diagnosis of ancient leprosy.

The observation that three cases from Winchester were infected with SNP type 3I-1 isolates is consistent with what is known of the geographical spread of the pathogen in prehistory and with earlier observations from human remains which have revealed the association of type 3 strains with Europe and Britain [12], [27]. We have previously reported a type 3I strain from a case in Suffolk, UK. This was burial 1914, excavated from Blackfriars friary, Ipswich [25], and dated between 1263 and 1538 AD. Detailed genotyping of this case showed a type 3I strain but with at least one nucleotide variation from typical “modern” 3I isolates (C at nucleotide position 7,614, [26]. This probably represents an intermediate, arguably earlier, genotype to extant 3I strains. The 3I isolates present in skeletons Sk2, Sk7 from Winchester showed SNP subsets typical of present-day strains (T at nucleotide 7,614). This has yet to be proven for Sk19 as genotyping of this locus failed, possibly due to poor DNA preservation or fragmentation of this region. With advances gleaned from additional comparative genomics [30] it was possible to show that the Winchester strains were typical of 3I-1 subtypes, having one copy of an 11 bp repeat sequence at inDel_17915 and a base G at nucleotide position 1,527,056 (compared to C, characteristic of the 3I-2 subtype). Although the three SNP type 3 cases exhibited the same subtype, it was possible to further resolve these using MLVA analysis with three loci, namely (AGA)20, (GTA)9 and 21-3 (Table 6). In our present analysis, it was found that the (AGA)20 microsatellite (initially described as the TTC triplet repeat) proved to be the more useful marker of these three for differentiating the Winchester cases, with Sk2, Sk7 and Sk19 showing 11, 13 and 14 copies of this motif respectively.

The remaining two fully genotyped cases, Sk8 and Sk14 were both found to be SNP type 2. Further genotyping with additional loci (1,104,235 and 3,102,787) showed these isolates displayed polymorphisms consistent with subtype F. To date this is the first description of type 2 isolates to be found in archaeological material from Britain. This group seems to be amongst the least common of extant strains. Type 2F has been reported in Turkey and Iran and other type 2 subtypes in Nepal (2G), Malawi (2E) and Ethiopia (2H) [27]. MLVA typing of isolates from Sk8 and Sk14 was not able to resolve the archaeological strains, which both showed the same 14-8-2 profile (Table 6). Therefore, if this were required, additional loci would need to be added to the analysis. A number of polymorphic VNTR loci have been identified and evaluated for studying short-term transmission chains and some of these are useful for archaeological applications [72].

There are relatively few successfully genotyped cases from the archaeological record for comparison. In addition to the type 3 cases from Europe mentioned above (mostly types K, L & M, [27], we have previously described a type 3L strain from a case of LL in Uzbekistan, Central Asia, dated to between the 1^st^ and 4^th^ centuries AD [26]. Suzuki and colleagues [73] have applied genotyping techniques to human remains excavated from Honshu Island in Japan. The remains in question were of a male skeleton, aged between 30–50 years at the time of death and dated to between the middle of the 18^th^ century to early 19^th^ century. Ancient DNA analysis showed the causative strain of *M. leprae* belonged to the main SNP type 1, which is the dominant group in India and Southeast Asia and which probably evolved from type 2 strains which had spread east from the Middle East.

Interestingly, type 2 strains have recently been described in medieval (10^th^-14^th^ centuries) cases of leprosy from Sigtuna, Sweden so that prevalence of these isolates in Europe may have been more common in the past [74]. The present study extends our knowledge of the westward spread of type 2 isolates into the British Isles, which had clearly occurred by the early medieval period.

Based on the number of nonsynonymous substitutions per site in pseudogenes, it has been estimated that a single pseudogenization event occurred in the leprosy bacillus in the last 10–20 million years [75]. Therefore, there is a huge interregnum between the estimated age of the pathogen since genome downsizing occurred and the known period in which it has caused disease in humans.

To date, the oldest leprosy skeletal remains found, from Balathal in India, are just 4000 years old [76]. Unfortunately, confirmation of the diagnosis by molecular methods, such as identification of *M. leprae* DNA, is not yet available for this specimen. However, such analyses are planned and any information resulting from them could be pivotal in understanding the early spread of disease out of Africa and establishing dates for the appearance of the predominant genotype associated with the Indian subcontinent.

### Conclusions

The preservation of remains excavated from the Winchester leprosy hospital site has allowed amplification of *M. leprae* DNA from nine individuals who showed osteological signs of multibacillary leprosy. Mycolic acid analysis independently confirmed the presence of leprosy in the same cases. In addition to demonstration of the leprosy pathogen in these individuals of early medieval period, genotyping of five cases showed two main strains, types, 3I-1 and type 2F. A type 3I strain has previously been reported in UK, but this is the first evidence for type 2 strains from this part of Europe. Type 2 strains are amongst the rarest of extant isolates and are probably closest to the most common recent ancestor of the leprosy clone which gave rise to the four main genotypes during global dissemination of the disease in prehistory. Whilst the present-day locations of type 2F strains include Turkey and Iran, the Sr isotopes of the three individuals studied are consistent with a childhood/adolescence on the Cretaceous chalk of southern Britain. The dietary isotopes do not show signs of an exotic diet, and together the evidence points to a local origin for all the individuals and suggests that 2F was an endemic strain in Britain at this time.
